# Distinct features between *MLH1*-methylated and unmethylated colorectal carcinomas with the CpG island methylator phenotype: implications in the serrated neoplasia pathway

**DOI:** 10.18632/oncotarget.7374

**Published:** 2016-02-13

**Authors:** Jung Ho Kim, Jeong Mo Bae, Nam-Yun Cho, Gyeong Hoon Kang

**Affiliations:** ^1^ Department of Pathology, Seoul National University Hospital, Seoul National University College of Medicine, Seoul, Korea; ^2^ Laboratory of Epigenetics, Cancer Research Institute, Seoul National University College of Medicine, Seoul, Korea

**Keywords:** colorectal cancer, CpG island methylator phenotype, microsatellite instability, MLH1, serrated pathway

## Abstract

The presence or absence of *MLH1* methylation may critically affect the heterogeneity of colorectal carcinoma (CRC) with the CpG island methylator phenotype (CIMP). Here, we investigated the differential characteristics of CIMP-high (CIMP-H) CRCs according to *MLH1* methylation status. To further confirm the *MLH1*-dependent features in CIMP-H CRC, an independent analysis was performed using data from The Cancer Genome Atlas (TCGA). In our CIMP-H CRC samples, *MLH1*-methylated tumors were characterized by older patient age, proximal colonic location, mucinous histology, intense lymphoid reactions, *RUNX3*/*SOCS1* promoter methylation, *BRAF* mutations, and microsatellite instability-high (MSI-H) status. By contrast, *MLH1*-unmethylated tumors were associated with earlier age of onset, increased distal colorectal localization, adverse pathologic features, and *KRAS* mutations. In the TCGA dataset, the *MLH1*-silenced CIMP-H CRC demonstrated proximal location, MSI-H status, hypermutated phenotype, and frequent *BRAF* mutations, but the *MLH1*-non-silenced CIMP-H CRC was significantly associated with high frequencies of *KRAS* and *APC* mutations. In conclusion, the differential nature of CIMP-H CRCs depends primarily on the *MLH1* methylation status. Based on the current knowledge, the sessile serrated adenoma/polyp may be the major precursor of *MLH1*-methylated CIMP-H CRCs, whereas *MLH1*-unmethylated CIMP-H CRCs may develop predominantly from *KRAS*-mutated traditional serrated adenomas and less commonly from *BRAF*-mutated traditional serrated adenomas and/or sessile serrated adenomas/polyps.

## INTRODUCTION

Major molecular pathways in colorectal carcinogenesis include the chromosomal instability (CIN) pathway, the microsatellite instability (MSI) pathway, and the CpG island methylator phenotype (CIMP) pathway [1-3]. MSI and CIMP are unique molecular phenotypes found in subsets of colorectal carcinoma (CRC) and have been revealed to be associated with characteristic clinicopathologic features and serrated precursor lesions [1-3]. MSI is caused by a defect in the DNA mismatch repair (MMR) system induced by a germline mutation or promoter methylation in one of the MMR genes (*MLH1, MSH2, MSH6,* and *PMS2)* or by a germline deletion at the 3′ end of the *EPCAM* gene [4-7]. MSI is characterized by vulnerability to length alterations in microsatellite DNA repeats throughout the genome, and therefore, it frequently results in mutations in many tumor-related genes [4-7]. CRC with MSI-high (MSI-H), which is considered the MSI-positive status, can occur in a hereditary (Lynch syndrome) or sporadic (CIMP-associated or serrated pathway-associated) setting [4-7]. By contrast to MSI, which is associated with genetic alterations, the CIMP is associated with epigenetic alterations in CRC and is characterized by extensive promoter CpG island hypermethylation and the subsequent transcriptional silencing of many tumor-related genes [8-10]. The majority of CRCs with CIMP-high (CIMP-H), which is considered the CIMP-positive status, are thought to develop sporadically from serrated precursor lesions [11, 12].

MSI-H has been reported to be associated with unique clinicopathologic and molecular features in CRC, including proximal colonic tumor location, mucinous histology, medullary histology, tumor-infiltrating lymphocytes, a peritumoral lymphoid reaction, a Crohn-like lymphoid reaction, the *BRAF* V600E mutation, resistance to 5-fluorouracil-based adjuvant chemotherapy, and a favorable prognosis [6]. The characteristics of CIMP-H CRC have also been investigated and are similar to those of MSI-H CRC. Representative CIMP-H-associated features in CRC are old age, poor prognosis, female sex, a proximal colonic tumor location, poorly differentiated histology, signet ring cell histology, serrated histology, the *BRAF* V600E mutation, and MSI-H status [8]. In fact, the significant overlap between MSI-H and CIMP-H in CRC is not surprising because nearly all sporadic MSI-H CRCs are molecularly based on the promoter CpG island hypermethylation-induced silencing of the *MLH1* gene, which is also found in a considerable number of CIMP-H CRCs. In this context, clear discrimination of CIMP-H-associated features from MSI-H-associated features in CRC might be difficult. Moreover, although *MLH1* methylation is regarded as one of the major molecular determinants of CIMP-H CRC, detailed features associated with *MLH1* methylation in CIMP-H CRC have not been fully elucidated.

Therefore, we aimed to comparatively and comprehensively investigate the differential clinicopathologic and molecular characteristics between *MLH1*-methylated tumors and *MLH1*-unmethylated tumors in CIMP-H CRC. In addition to analysis of CRC samples, independent statistical analysis of *MLH1* silencing-associated features in CIMP-H CRC was conducted using The Cancer Genome Atlas (TCGA) data. Through these analyses, we expected to more precisely determine the *MLH1* methylation-dependent clinicopathologic and molecular heterogeneity of CIMP-H CRC and to obtain a deeper understanding of the potential connection between CIMP-H CRC and serrated precursor pathways.

## RESULTS

### Distinct clinicopathologic features according to *MLH1* methylation status in CIMP-H CRCs

The differential clinicopathologic features of our study samples (65 CIMP-H CRCs) according to *MLH1* promoter methylation status are summarized in Table 1. CIMP-H CRC with *MLH1* methylation (*n* = 33) was significantly associated with old age (66 years or older; *P* < 0.001), a proximal colonic tumor location (82%; *P* = 0.026), extracellular mucinous histology (73%; *P* < 0.001), high-density tumor-infiltrating lymphocytes (TILs) (58%; *P* < 0.001), moderate to marked peritumoral lymphoid reaction (53%; *P* = 0.002), and active (34%; *P* = 0.005) and high-density (69%; *P* = 0.024) Crohn-like lymphoid reaction (Table 1). By contrast, CIMP-H CRC without *MLH1* methylation (*n* = 32) demonstrated significantly higher frequencies of distant metastasis (34%; *P* = 0.033), vascular invasion (28%; *P* = 0.006), perineural invasion (47%; *P* = 0.006), and tumor budding (81%; *P* = 0.011) (Table 1).

**Table 1:** Differential clinicopathologic features of CIMP-H CRC according to *MLH1* promoter methylation status (original study samples; *n* = 65)

Variables		Case No.	*MLH1*-methylated (*n* = 33)	*MLH1*-unmethylated (*n* = 32)	*P*-value
Age^a^	< 66 years	27	5 (15%)	22 (69%)	< 0.001
	≥ 66 years	38	28 (85%)	10 (31%)	
Sex	Male	32	15 (45%)	17 (53%)	0.536
	Female	33	18 (55%)	15 (47%)	
Tumor location	Proximal	45	27 (82%)	18 (56%)	0.026
	Distal	20	6 (18%)	14 (44%)	
Gross tumor type	Fungating	39	20 (61%)	19 (59%)	0.919
	Infiltrative	26	13 (39%)	13 (41%)	
AJCC TNM stage	Stage I/II	20	12 (36%)	8 (25%)	0.321
	Stage III/IV	45	21 (64%)	24 (75%)	
Depth of primary tumor invasion (pT category)	pT1/pT2	2	1 (3%)	1 (3%)	1
	pT3/pT4	63	32 (97%)	31 (97%)	
Lymph node metastasis (pN category)	Absent (pN0)	20	12 (36%)	8 (25%)	0.321
	Present (pN1/pN2)	45	21 (64%)	24 (75%)	
Distant metastasis (M category)	Absent (M0)	50	29 (88%)	21 (66%)	0.033
	Present (M1)	15	4 (12%)	11 (34%)	
Tumor differentiation	WD/MD	48	23 (70%)	25 (78%)	0.44
	PD	17	10 (30%)	7 (22%)	
Mucinous histology	Absent	34	9 (27%)	25 (78%)	< 0.001
	Present	31	24 (73%)	7 (22%)	
Signet ring cell histology	Absent	58	27 (82%)	31 (97%)	0.105
	Present	7	6 (18%)	1 (3%)	
Medullary histology	Absent	60	29 (88%)	31 (97%)	0.355
	Present	5	4 (12%)	1 (3%)	
Serrated histology	Absent	50	26 (79%)	24 (75%)	0.717
	Present	15	7 (21%)	8 (25%)	
Lymphatic invasion	Absent	22	12 (36%)	10 (31%)	0.663
	Present	43	21 (64%)	22 (69%)	
Venous invasion	Absent	55	32 (97%)	23 (72%)	0.006
	Present	10	1 (3%)	9 (28%)	
Perineural invasion	Absent	45	28 (85%)	17 (53%)	0.006
	Present	20	5 (15%)	15 (47%)	
Tumor budding	Absent	22	16 (48%)	6 (19%)	0.011
	Present	43	17 (52%)	26 (81%)	
Tumor-infiltrating lymphocytes (TILs)^b^	Low-density (< 21 TILs/HPF)	46	14 (42%)	32 (100%)	< 0.001
	High-density (≥ 21 TILs/HPF)	19	19 (58%)	0 (0%)	
Peritumoral lymphoid reaction^b^	Absent or mild	42	15 (47%)	27 (84%)	0.002
	Moderate to marked	22	17 (53%)	5 (16%)	
Crohn-like lymphoid reaction (Ueno criteria)^c^	Inactive (largest LA < 1 mm)	51	21 (66%)	30 (94%)	0.005
	Active (largest LA ≥ 1 mm)	13	11 (34%)	2 (6%)	
Crohn-like lymphoid reaction (Väyrynen-Mäkinen criteria)^c^	Low-density (< 0.38/mm)	29	10 (31%)	19 (59%)	0.024
	High-density (≥ 0.38/mm)	35	22 (69%)	13 (41%)	

Abbreviations: CIMP-H, CpG island methylator phenotype-high; AJCC, American Joint Committee on Cancer; TNM, tumor-node-metastasis; WD, well differentiated; MD, moderately differentiated; PD, poorly differentiated; HPF, high power field; LA, lymphoid aggregate.

a Dichotomous age groups were classified using a cutoff value of the average age (66 years).

b Dichotomous TIL groups were classified using a cutoff value of the average TIL density (21/HPF).

c Peritumoral lymphoid reaction and Crohn-like lymphoid reaction were assessed in 64 out of the 65 CIMP-H CRCs due to the inadequate quality of tissue sections for lymphoid reaction evaluations in one case.

**Figure 1:**
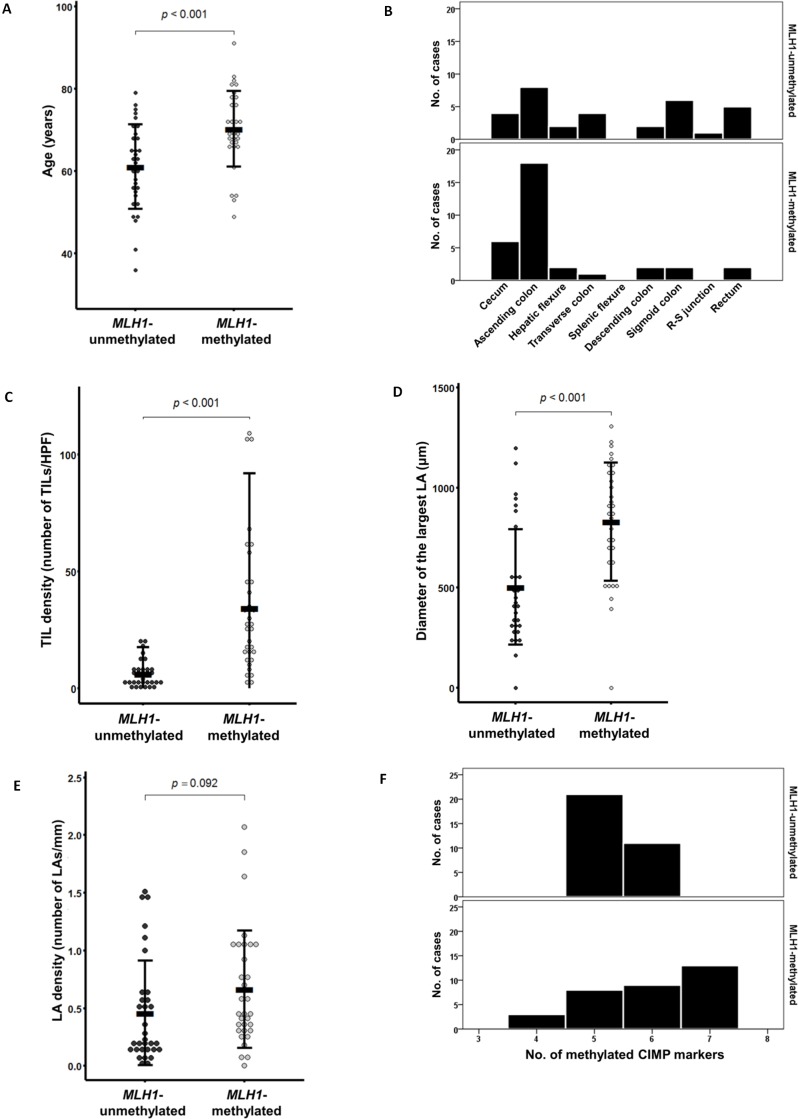
Comparison of quantitative clinicopathologic parameters according to *MLH1* promoter methylation status in CIMP-H CRC (*n* = 65) **A.** Age at diagnosis. **B.** Tumor location bowel subsite. **C.** Tumor-infiltrating lymphocyte density (number of tumor-infiltrating lymphocytes/high power field). **D.** Size of the largest Crohn-like lymphoid aggregate (μm). **E.** Crohn-like lymphoid reaction density (number of lymphoid aggregates/mm). **F.** Number of methylated CIMP markers (except for *MLH1*). Abbreviations: TIL, tumor-infiltrating lymphocyte; HPF, high power field; LA, lymphoid aggregate.

Further detailed analyses taking into account age and tumor location were performed. The age distribution of the *MLH1*-unmethylated tumors (mean age ± standard deviation = 61.1 ± 10.3 years; age range = 36 to 79 years) was shifted more toward middle age compared with that of the *MLH1*-methylated tumors (mean age ± standard deviation = 70.2 ± 9.2 years; age range = 49 to 91 years) (*P* < 0.001; Figure 1A). A comparison of the bowel subsite distribution of tumor location between the *MLH1*-methylated and *MLH1*-unmethylated tumors is shown in Figure 1B. The most prevalent bowel subsite of the *MLH1*-methylated tumors was the ascending colon (55%), followed by the cecum (18%) (Figure 1B). By contrast, although the *MLH1*-unmethylated tumors were frequently located in the ascending colon (25%), the sigmoid colon and rectum were also major bowel subsites (19% and 16%, respectively) (Figure 1B).

Next, quantitatively measured raw data on TILs and Crohn-like lymphoid reactions were further analyzed. The mean value of the density of TILs in the *MLH1*-methylated tumors (mean density ± standard deviation = 34.4 ± 29.3 TILs/high power field) was significantly higher than that in the *MLH1*-unmethylated tumors (mean density ± standard deviation = 6.3 ± 5.8 TILs/high power field) (*P* < 0.001; Figure 1C). The mean value of the diameter of the largest lymphoid aggregate in the *MLH1*-methylated tumors (mean diameter ± standard deviation = 830.6 ± 295.3 μm) was also significantly higher than that in the *MLH1*-unmethylated tumors (mean diameter ± standard deviation = 503.5 ± 288.1 μm) (*P* < 0.001; Figure 1D). A comparison of lymphoid aggregate density between the *MLH1*-methylated (mean density ± standard deviation = 0.66 ± 0.51 lymphoid aggregates/mm) and *MLH1*-unmethylated tumors (mean density ± standard deviation = 0.46 ± 0.45 lymphoid aggregates/mm) is shown in Figure 1E.

**Figure 2:**
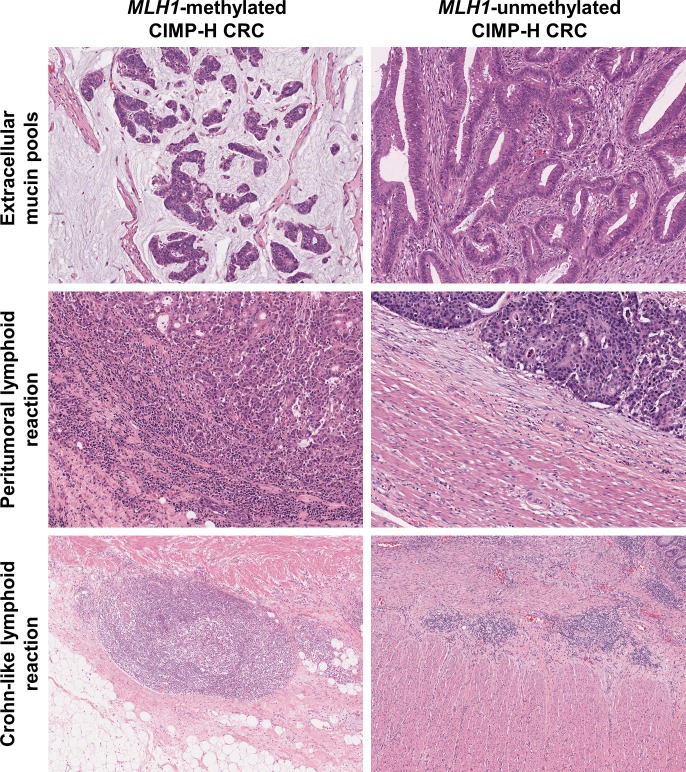
Differential histopathologic features according to *MLH1* promoter methylation status in CIMP-H CRC (**Upper left**) Typical mucinous histology in a case of *MLH1*-methylated CIMP-H CRC (H&E, ×100). (**Upper right**) Non-mucinous adenocarcinoma in a case of *MLH1*-unmethylated CIMP-H CRC (H&E, ×100). (**Middle left**) Intense peritumoral lymphoid reaction in a case of *MLH1*-methylated CIMP-H CRC (H&E, ×100). (**Middle right**) Absent peritumoral lymphoid reaction in a case of *MLH1*-unmethylated CIMP-H CRC (H&E, ×100). (**Lower left**) Active Crohn-like lymphoid reaction in a case of *MLH1*-methylated CIMP-H CRC. Note the large (> 1 mm) lymphoid aggregate (H&E, ×50). (**Lower right**) Inactive Crohn-like lymphoid reaction in a case of *MLH1*-unmethylated CIMP-H CRC. Note the small (< 1 mm) lymphoid aggregates (H&E, ×50).

Representative photomicrographs of the *MLH1* methylation-dependent differential histopathologic features in our CIMP-H CRC samples are shown in Figure 2. The *MLH1*-methylated tumors frequently exhibited abundant mucin pools, intense peritumoral lymphoid reactions, and active Crohn-like lymphoid reactions, whereas the *MLH1*-unmethylated tumors were associated with non-mucinous-type adenocarcinoma and the absence of peritumoral lymphoid reaction and Crohn-like lymphoid reaction (Figure 2).

The *MLH1*-unmethylated CIMP-H CRCs showed a tendency toward worse disease-free survival compared with the *MLH1*-methylated CIMP-H CRCs, as shown by Kaplan-Meier survival analysis; however, this survival difference was not statistically significant (log-rank *P* = 0.438; Supplementary Figure S1A). In addition, prognostic significance of *MLH1* methylation was not observed in 45 CIMP-H CRCs treated with 5-fluorouracil-based adjuvant chemotherapy (log-rank *P* = 0.157; Supplementary Figure S1B).

### Distinct molecular features according to *MLH1* methylation status in CIMP-H CRCs

The differential molecular features of 65 CIMP-H CRCs according to *MLH1* promoter methylation status are summarized in Table 2. As expected, most of the *MLH1*-methylated CIMP-H CRCs demonstrated MSI-H status (94%; *P* < 0.001; Table 2). The *MLH1*-methylated tumors were also associated with a relatively high frequency of the *BRAF* V600E mutation (36%; *P* = 0.03), whereas the *MLH1*-unmethylated tumors were significantly correlated with *KRAS* mutations (38%; *P* = 0.001) (Table 2).

**Table 2:** Differential molecular features of CIMP-H CRC according to *MLH1* promoter methylation status (original study samples; n = 65)

Variables		Case No.	*MLH1*-methylated (*n* = 33)	*MLH1*-unmethylated (*n* = 32)	*P*-value
MSI status	MSI-L/MSS	29	2 (6%)	27 (84%)	< 0.001
	MSI-H	36	31 (94%)	5 (16%)	
No. of methylated CIMP markers (except for *MLH1*)	< 6	32	11 (33%)	21 (66%)	0.009
	≥ 6	33	22 (67%)	11 (34%)	
*CRABP1* methylation	Methylated	64	32 (97%)	32 (100%)	1
	Unmethylated	1	1 (3%)	0 (0%)	
*CDKN2A* (*p16*) methylation	Methylated	62	31 (94%)	31 (97%)	1
	Unmethylated	3	2 (6%)	1 (3%)	
*CACNA1G* methylation	Methylated	64	32 (97%)	32 (100%)	1
	Unmethylated	1	1 (3%)	0 (0%)	
*NEUROG1* methylation	Methylated	60	30 (91%)	30 (94%)	1
	Unmethylated	5	3 (9%)	2 (6%)	
*IGF2* methylation	Methylated	62	32 (97%)	30 (94%)	0.613
	Unmethylated	3	1 (3%)	2 (6%)	
*RUNX3* methylation	Methylated	34	22 (67%)	12 (38%)	0.019
	Unmethylated	31	11 (33%)	20 (63%)	
*SOCS1* methylation	Methylated	22	18 (55%)	4 (13%)	< 0.001
	Unmethylated	43	15 (45%)	28 (88%)	
*KRAS* mutation^a^	Mutant	12	1 (3%)	11 (38%)	0.001
	Wild type	49	31 (97%)	18 (62%)	
*BRAF* mutation^b^	Mutant	16	12 (36%)	4 (13%)	0.03
	Wild type	48	21 (64%)	27 (87%)	

Abbreviations: CIMP-H, CpG island methylator phenotype-high; MSI, microsatellite instability; MSI-L, MSI-low; MSI-H, MSI-high; MSS, microsatellite stable.

a *KRAS* mutation analysis was performed on 61 out of the 65 CIMP-H CRCs due to a limited amount or suboptimal quality of extracted tumor DNA in four cases.

b *BRAF* mutation analysis was performed on 64 out of the 65 CIMP-H CRCs due to a limited amount or suboptimal quality of extracted tumor DNA in one case.

Notably, the *MLH1*-methylated tumors were associated with more extensive CpG island methylation compared with the *MLH1*-unmethylated tumors. Twenty-two of the 33 *MLH1*-methylated tumors (67%) had six or seven methylated markers among the seven CIMP markers (except for *MLH1*) (Table 2 and Figure 1F). By contrast, 21 of the 32 *MLH1*-unmethylated tumors (66%) demonstrated five methylated CIMP markers (Table 2 and Figure 1F). This difference in the number of methylated CIMP markers between the *MLH1*-methylated and *MLH1*-unmethylated tumors was based primarily on differences in the methylation frequencies of two CIMP markers, *RUNX3* and *SOCS1*. The *MLH1*-methylated tumors showed significantly higher frequencies of promoter methylation of *RUNX3* (67%; *P* = 0.019) and *SOCS1* (55%; *P* < 0.001) (Table 2).

### TCGA data analysis

**Figure 3:**
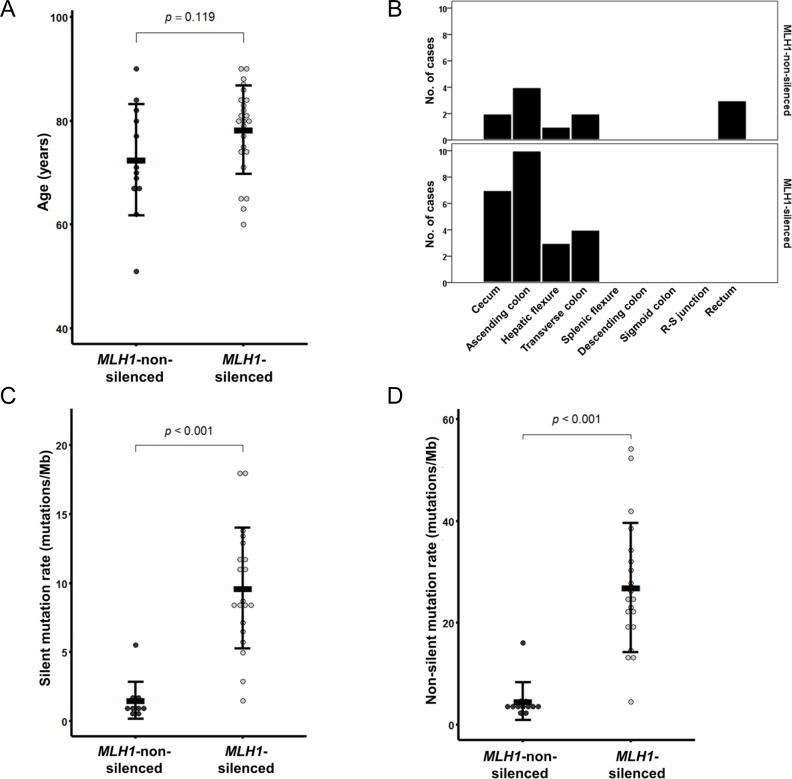
*MLH1* silencing-associated features in CIMP-H CRC from the TCGA dataset (*n* = 36) **A.** Age at diagnosis. **B.** Tumor location bowel subsite. **C.** Silent mutation rate. **D.** Non-silent mutation rate.

From the TCGA dataset, a total of 36 CIMP-H CRCs were identified (24 *MLH1*-silenced and 12 *MLH1*-non-silenced tumors). Of these, the *MLH1*-silenced tumors were significantly associated with female sex (79%; *P* = 0.003), proximal colonic tumor location (100%; *P* = 0.031), MSI-H status (96%; *P* < 0.001), an exome-wide hypermutated phenotype (95%; *P* < 0.001), wild-type *KRAS* (95%; *P* < 0.001), and mutated *BRAF* (75%; *P* < 0.001) (Table 3). Characteristically, *KRAS* and *APC* mutations were predominantly observed in the *MLH1*-non-silenced tumors (each 83%; each *P* < 0.001; Table 3). Compared with the *MLH1*-non-silenced tumors, the *MLH1*-silenced tumors showed a tendency toward old age at diagnosis (mean age ± standard deviation = 78.3 ± 8.5 years; age range = 60 to 90 years; Figure 3A and Table 3) and mucinous histology (43%; Table 3), although these findings were not statistically significant. Two of the *MLH1*-non-silenced tumors were distant metastatic tumors (17%; Table 3) and three were rectal tumors (25%; Table 3 and Figure 3B), whereas the *MLH1*-silenced tumors showed no distant metastasis or distal colorectal localization (Table 3 and Figure 3B). Detailed analysis of the genomic mutation rate revealed that the *MLH1*-silenced tumors had significantly higher rates of both silent mutations (mean mutation rate ± standard deviation = 9.6 ± 4.4 mutations/Mb; range = 1.5 to 18.1 mutations/Mb; *P* < 0.001; Figure 3C) and non-silent mutations (mean mutation rate ± standard deviation = 26.9 ± 12.7 mutations/Mb; range = 4.5 to 54.2 mutations/Mb; *P* < 0.001; Figure 3D).

**Table 3:** Differential features of CIMP-H CRC according to *MLH1* silencing status (TCGA dataset; *n* = 36)

Variables		Case No.	*MLH1*-silenced (*n* = 24)	*MLH1*-non-silenced (*n* = 12)	*P*-value
Age^a^	< 76 years	15	8 (33%)	7 (58%)	0.151
	≥ 76 years	21	16 (67%)	5 (42%)	
Sex	Male	14	5 (21%)	9 (75%)	0.003
	Female	22	19 (79%)	3 (25%)	
Tumor location	Proximal	33	24 (100%)	9 (75%)	0.031
	Distal	3	0 (0%)	3 (25%)	
AJCC TNM stage^b^	Stage I/II	23	16 (70%)	7 (58%)	0.709
	Stage III/IV	12	7 (30%)	5 (42%)	
Depth of primary tumor invasion (pT category)^c^	pT1/pT2	5	3 (16%)	2 (18%)	1
	pT3/pT4	25	16 (84%)	9 (82%)	
Lymph node metastasis (pN category)	Absent (pN0)	24	17 (71%)	7 (58%)	0.479
	Present (pN1/pN2)	12	7 (29%)	5 (42%)	
Distant metastasis (M category)	Absent (M0)	34	24 (100%)	10 (83%)	0.105
	Present (M1)	2	0 (0%)	2 (17%)	
Histologic subtype^d^	Non-mucinous	22	13 (57%)	9 (75%)	0.463
	Mucinous	13	10 (43%)	3 (25%)	
Lymphatic invasion^e^	Absent	17	13 (57%)	4 (40%)	0.465
	Present	16	10 (43%)	6 (60%)	
Vascular invasion^f^	Absent	22	14 (78%)	8 (89%)	0.636
	Present	5	4 (22%)	1 (11%)	
MSI status	MSI-L/MSS	12	1 (4%)	11 (92%)	< 0.001
	MSI-H	24	23 (96%)	1 (8%)	
Mutational phenotype^g^	Non-hypermutated	12	1 (5%)	11 (92%)	< 0.001
	Hypermutated	20	19 (95%)	1 (8%)	
*KRAS* mutation^h^	Mutant	11	1 (5%)	10 (83%)	< 0.001
	Wild type	21	19 (95%)	2 (17%)	
*BRAF* mutation^h^	Mutant	16	15 (75%)	1 (8%)	< 0.001
	Wild type	16	5 (25%)	11 (92%)	
*PIK3CA* mutation^h^	Mutant	9	4 (20%)	5 (42%)	0.24
	Wild type	23	16 (80%)	7 (58%)	
*APC* mutation^h^	Mutant	14	4 (20%)	10 (83%)	< 0.001
	Wild type	18	16 (80%)	2 (17%)	
*CTNNB1* mutation^h^	Mutant	1	0 (0%)	1 (8%)	0.375
	Wild type	31	20 (100%)	11 (92%)	
*TP53* mutation^h^	Mutant	7	3 (15%)	4 (33%)	0.379
	Wild type	25	17 (85%)	8 (67%)	

Abbreviations: CIMP-H, CpG island methylator phenotype-high; AJCC, American Joint Committee on Cancer; TNM, tumor-node-metastasis; MSI, microsatellite instability; MSI-L, MSI-low; MSI-H, MSI-high; MSS, microsatellite stable.

a Dichotomous age groups were classified using a cutoff value of the average age (76 years).

b AJCC TNM stage data were not available in one case among the 36 CIMP-H CRCs from the TCGA dataset.

c Depth of primary tumor invasion (pT) data were not available in six cases among the 36 CIMP-H CRCs from the TCGA dataset.

d Histologic subtype (mucinous adenocarcinoma) data were not available in one case among the 36 CIMP-H CRCs from the TCGA dataset.

e Lymphatic invasion data were not available in three cases among the 36 CIMP-H CRCs from the TCGA dataset.

f Vascular invasion data were not available in nine cases among the 36 CIMP-H CRCs from the TCGA dataset.

g Mutation rate data were not available in four cases among the 36 CIMP-H CRCs from the TCGA dataset.

h *KRAS*/*BRAF*/*PIK3CA*/*APC*/*CTNNB1*/*TP53* mutations data were not available in four cases among the 36 CIMP-H CRCs from the TCGA dataset.

## DISCUSSION

The presence or absence of *MLH1* methylation is a critical determinant of two distinct molecular subtypes of CIMP-H CRC: MSI-positive (MSI-H) and MSI-negative (MSS or MSI-L) CIMP-H CRCs. According to previous findings, the development of MSI-H status in a tumor genome is thought to be a late event that occurs in the serrated neoplasia pathway: although it is frequently found in combination with CIMP-H CRC, the majority of serrated precursor lesions, including sessile serrated adenomas/polyps (SSA/Ps) and traditional serrated adenomas (TSAs), have an MSS or MSI-L status [13]. Thus, *MLH1* promoter methylation and the subsequent acquisition of the MSI-H molecular phenotype may preferentially occur at the advanced dysplastic stage and/or the initiation of malignant transformation of serrated lesions. MSI-H status leads to genomic vulnerability associated with accumulation of mutations and neoplastic progression, finally inducing development of the unique clinicopathologic features of CRC. Representative MSI-H-associated features in CRC include frequent frameshift mutations, prominent intratumoral and peritumoral lymphocytic reactions, and favorable prognosis [6, 7, 14]. In our present study, to display the heterogeneous landscape of CIMP-H CRC according to *MLH1* methylation status, which critically determines MSI status, we analyzed various clinicopathologic and molecular factors as well as known MSI-associated features in CIMP-H CRC. As a result, we successfully obtained comprehensive data regarding *MLH1* methylation-dependent characteristics in CIMP-H CRC and their implications in different serrated precursor pathways.

Both histopathologic and prognostic features associated with *MLH1* methylation can be regarded as being directly related to MSI status in CIMP-H CRC. In the current study, *MLH1*-methylated tumors were found to be significantly associated with dense TILs, intense peritumoral lymphoid reactions and active/dense Crohn-like lymphoid reactions (Table 1 and Figures 1 and 2). By contrast, most histopathologic factors that are known to be associated with poor prognosis, including vascular invasion, perineural invasion, tumor budding, and distant metastasis, were observed more frequently in *MLH1*-unmethylated tumors than in *MLH1*-methylated tumors (Table 1). Moreover, although statistical significance was not found, a tendency toward better survival was observed in the *MLH1*-methylated tumors (Supplementary Figures S1A and S1B). All of these findings are consistent with the expected effects of MSI-H status in CRC. Because a significant association between anti-tumor immune reactions and improved patient survival has been demonstrated in diverse human malignancies, including CRC [15], it is reasonable to infer that the favorable prognostic impact of MSI-H in CRC may be mainly due to accompanying prominent lymphoid reactions [14]. Several previous studies have explained the potential mechanism underlying MSI-induced lymphoid reactions in CRC. Schwitalle et al. demonstrated that many tumor-specific frameshift peptides are generated in MSI-H CRC and that these neoantigens cause increased anti-tumor immune responses [16]. A recent investigation also revealed that the number of frameshift mutations is positively correlated with the density of T cell infiltrates in MSI-H CRC [17].

Mucinous differentiation has been shown to be associated with MSI-H, CIMP-H, *BRAF* mutation, and proximal colonic tumor location in CRC [18]. In the present study, *MLH1*-methylated CIMP-H CRCs were significantly correlated with a high frequency of mucinous histology (Table 1 and Figure 2). This finding suggests that extracellular mucin production might be molecularly related to MSI-H rather than to CIMP-H in CRC. Because the *MLH1*-methylated CIMP-H CRCs were concentrated in the proximal colon, to exclude the possibility of a tumor location effect on mucinous histology, we additionally performed subgroup analyses according to tumor location. The results demonstrated that in both the proximal colon and distal colorectum, the *MLH1*-methylated CIMP-H CRCs showed significantly more mucinous differentiation than the *MLH1*-unmethylated CIMP-H CRCs (Supplementary Tables S1 and S2). Furthermore, in an independent analysis using our extended CRC cohort (*n* = 687), we found that mucinous differentiation was more frequent in MSI-H, CIMP-low/negative (CIMP-L/0) CRC than in MSS/MSI-L, CIMP-L/0 CRC (36% and 9%, respectively; *P* < 0.001; Supplementary Table S3). Therefore, regardless of the proximal or distal tumor location and CIMP status, MSI-H can be regarded as a significant molecular predisposing factor for extracellular mucinous histology in CRC. In addition, because the frequency of mucinous histology was remarkably higher in *MLH1*-methylated CIMP-H CRC (73%; Table 1) than in MSI-H, CIMP-L/0 CRC (36%; Supplementary Table S3), there may be a synergistic impact of MSI-H and CIMP-H on mucinous differentiation in CRC.

**Figure 4:**
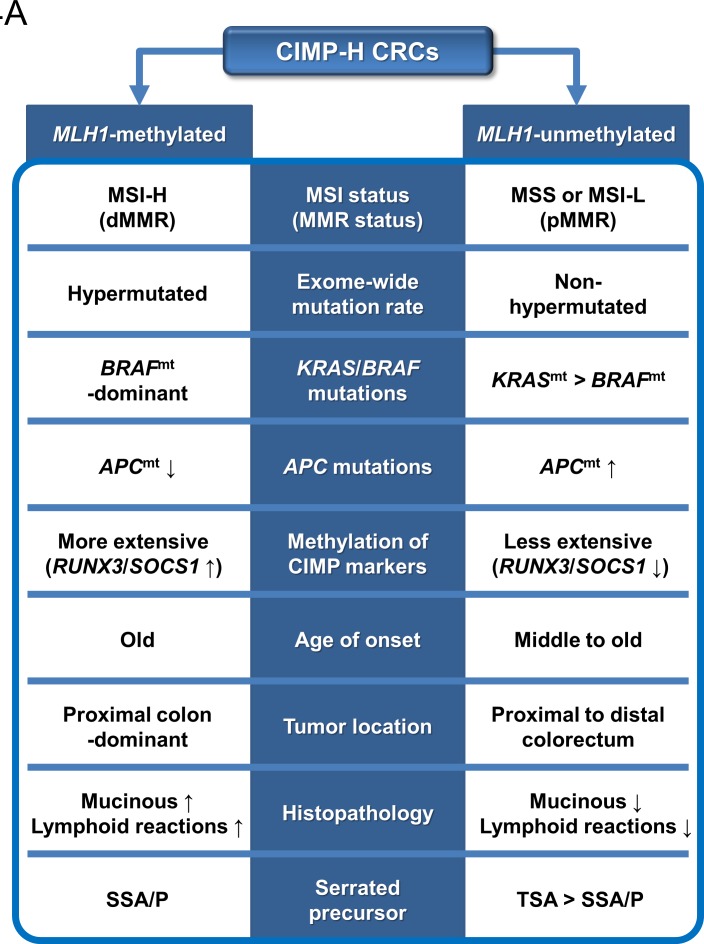

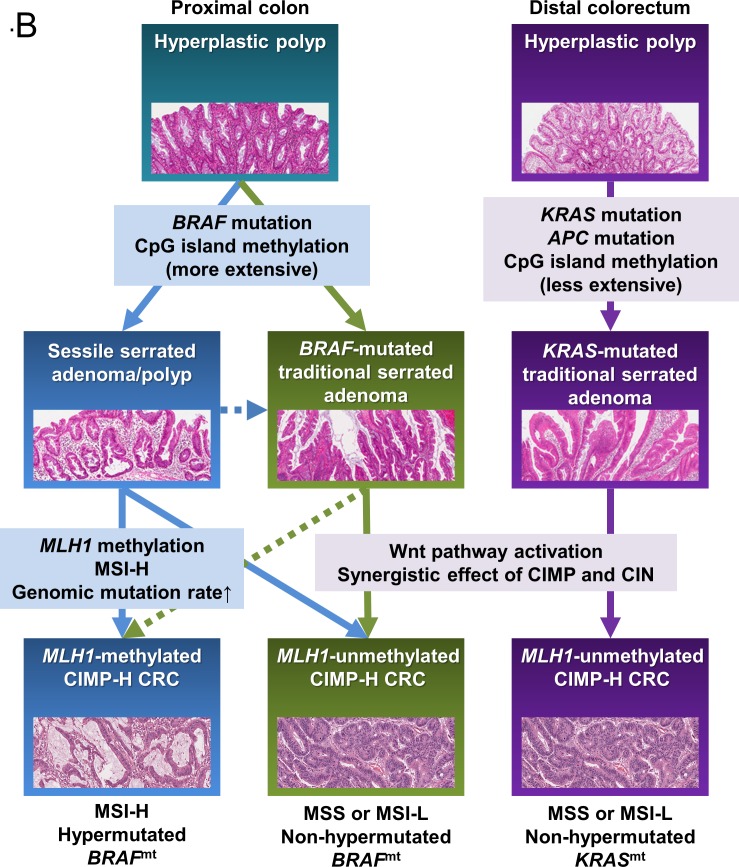
Schematic summary of this study **A.** Distinct features and precursors of the *MLH1*-methylated and unmethylated CIMP-H CRCs. **B.** A molecular pathogenetic model of CIMP-H CRCs based on different serrated precursor pathways. Abbreviations: CIMP-H, CpG island methylator phenotype-high; MSI, microsatellite instability; MSI-H, MSI-high; MSI-L, MSI-low; MSS, microsatellite stable; MMR, DNA mismatch repair; dMMR, MMR-deficient; pMMR, MMR-proficient; *BRAF*^mt^, *BRAF* mutation or *BRAF*-mutated; *KRAS*^mt^, *KRAS* mutation or *KRAS*-mutated; *APC*^mt^, *APC* mutation; SSA/P, sessile serrated adenoma/polyp; TSA, traditional serrated adenoma; CIN, chromosomal instability.

The differential clinicopathologic and molecular features between *MLH1*-methylated and *MLH1*-unmethylated CIMP-H CRCs provide important clues regarding the major precursor lesions of CIMP-H CRC. Intensive investigations conducted in the past decade have revealed that the majority of CIMP-H CRCs develop from serrated precursor lesions, especially from SSA/Ps [12]. However, our results indicate that substantial proportions of *MLH1*-methylated and *MLH1*-unmethylated CIMP-H CRCs may be derived from different subtypes of serrated lesions. Fundamentally, although the data are variable across studies, previous investigations have reported that the *MLH1* methylation frequency is higher in SSA/Ps (16% to 75%) than in TSAs (3% to 48%) [19]. Previous studies have also revealed that SSA/Ps are significantly associated with a proximal colonic tumor location, *BRAF* mutations, and CIMP-H status, whereas TSAs show relatively heterogeneous clinicopathologic and molecular features [12]. Therefore, the close relationship between SSA/Ps and *MLH1*-methylated CIMP-H CRC can be easily inferred, but a detailed approach is needed to identify the link between TSA and CIMP-H CRC. Fortunately, recent advances in the characterization of TSA suggest that it can be classified into three distinct molecular subtypes: *KRAS*-mutated, *BRAF*-mutated, and *KRAS*/*BRAF*-wild type [20-23]. Importantly, compared with *KRAS*-mutated TSA, *BRAF*-mutated TSA is significantly associated with a proximal colonic tumor location and more widespread CpG island methylation [20, 21]. According to a study by Bettington et al., *MLH1* methylation occurs in *BRAF*-mutated TSA but not in *KRAS*-mutated TSA, although the frequency is low (7% of *BRAF*-mutated TSAs) [20]. In addition, Wiland et al. detected *RUNX3* and *SOCS1* methylation in *BRAF*-mutated TSA but not in *KRAS*-mutated TSA [21]. In our study cohort, *BRAF*-mutated, *MLH1*-unmethylated CIMP-H CRCs generally showed more extensive methylation of CIMP markers than *KRAS*-mutated, *MLH1*-unmethylated CIMP-H CRCs (Supplementary Figure S2). These features imply that *BRAF* mutation is tightly associated with extensive CpG island methylation in colorectal carcinogenesis, regardless of whether the carcinogenesis pathway is the SSA/P pathway or the TSA pathway (Figure 4A and 4B).

According to our findings, *MLH1*-methylated CIMP-H CRC was significantly associated with high frequencies of a proximal colonic tumor location and *RUNX3*/*SOCS1* promoter methylation compared with *MLH1*-unmethylated CIMP-H CRC (Tables 1, 2). On the basis of comprehensive profiles of proximal/distal tumor locations, *KRAS*/*BRAF* mutations, and *MLH1*/*RUNX3*/*SOCS1* methylation of serrated lesions, the major precursor of *MLH1*-methylated CIMP-H CRC may be SSA/P, whereas the major precursors of *MLH1*-unmethylated CIMP-H CRC may be *KRAS*-mutated TSA, followed by *BRAF*-mutated TSA and SSA/P (Figure 4A and 4B).

According to our data, although *KRAS* mutations were the dominant form of *RAS*/*RAF* pathway mutations in *MLH1*-unmethylated CIMP-H CRC, a small portion of *MLH1*-unmethylated CIMP-H CRCs displayed *BRAF* mutations (8% to 13%; Tables 2, 3). Therefore, the minority of *MLH1*-unmethylated CIMP-H CRCs, which have *BRAF* mutations, may develop from SSA/P and/or *BRAF*-mutated TSA (Figure 4B). In our samples, *BRAF*-mutated, *MLH1*-unmethylated CIMP-H CRC (*n* = 4) was associated with aggressive clinicopathologic features (Supplementary Table S4). All four cases of *BRAF*-mutated, *MLH1*-unmethylated tumor histologically showed perineural invasion and tumor budding-positivity. Of the four cases, two patients were stage IV and died within 4 months after surgical treatment. Another patient was stage III, and cancer recurred at 19.5 months after surgical treatment. These findings were consistent with the results of a study by Pai et al., who demonstrated that *BRAF*-mutated, MSS-type proximal colonic adenocarcinoma was associated with adverse histologic features and poor survival [24]. Collectively, a small subset of *MLH1*-unmethylated CIMP-H CRCs may be derived from *BRAF*-mutated TSA and/or SSA/P (Figure 4B), and these *BRAF*-mutated, *MLH1*-unmethylated tumors constitute a highly aggressive phenotype among CRCs.

In terms of the age distribution of CIMP-H CRCs, we found that older patients had *MLH1*-methylated tumors more frequently than they had *MLH1*-unmethylated tumors (Table 1 and Figure 1a). However, although advanced SSA/P is known to be prevalent in old females [25], there are no established data showing a significant age difference between individuals with SSA/P and TSA. Therefore, a hypothetical theory can be suggested that is based on different rates of dysplastic change and/or different chances of malignant transformation between SSA/P and TSA. SSA/P (4% to 23% of all serrated lesions) is known to be more frequent than TSA (1% to 2% of all serrated lesions) [19]. However, advanced SSA/P and its malignant counterpart, *MLH1*-methylated CIMP-H CRC, occur at an older age than advanced TSA and *MLH1*-unmethylated CIMP-H CRC. These features imply that dysplastic change and the malignant transformation of SSA/P may be more slow and rare than those of TSA. This presumed biological difference in neoplastic progression between SSA/P and TSA might be based on the underlying molecular differences. In fact, *KRAS*-mutated TSA has been regarded as an aggressive serrated lesion that is significantly associated with high-grade dysplasia and intramucosal adenocarcinoma and is frequently found in combination with *MGMT* and *CDKN2A* methylation, *TP53* mutations, and Wnt pathway activation, suggestive of the combined activity of the CIMP and CIN pathways [23, 26]. Moreover, a recent study by Panarelli et al. reported a lack of evidence of Wnt pathway activation in sporadic MSI-H CRC [27]. Our results from TCGA data analysis also demonstrated that *MLH1*-non-silenced CIMP-H CRC showed frequent *APC* mutations, but *MLH1*-silenced CIMP-H CRC did not (83% *vs*. 20%; Table 3). These findings support that in contrast with the TSA pathway, which may partly depend on *APC* mutation-associated conventional adenoma-carcinoma pathway, the SSA/P pathway may be molecularly associated with the pure CIMP pathway (Figure 4B). Therefore, it can be hypothesized that the synergistic fusion of molecular pathways in TSA may contribute to the relatively rapid and frequent progression of low-grade dysplasia to high-grade dysplasia and carcinoma and may ultimately underlie the age difference between patients with *MLH1*-methyated and *MLH1*-unmethylated CIMP-H CRCs.

In this study, to identify distinct features between *MLH1*-methylated and unmethylated CIMP-H CRCs, we analyzed both our original cohort data and the TCGA data. We successfully confirmed that the majority of differential features between *MLH1*-methylated and unmethylated tumors identified from our cohort data was also maintained in results from the TCGA data analysis. However, underlying characteristics of our samples and TCGA samples were somewhat different, which representatively included *BRAF* mutation frequency. In our cohort, only 36% of *MLH1*-methylated CIMP-H CRCs harbor *BRAF* mutations, whereas *BRAF* mutation frequency of *MLH1*-silenced CIMP-H CRCs in the TCGA data was 75%. This discrepancy of *BRAF* mutation frequency may be due to the ethnic difference of molecular features in CRCs. According to our previous investigation, frequencies of *BRAF* mutation and CIMP-H in East Asian patients with CRC were significantly lower than those in Western country patients with CRC [6]. In fact, our cohort was comprised exclusively of East Asians, but the racial composition of the TCGA cohort was heterogeneous. This difference of study populations can induce differences in molecular features of CRCs, especially in *BRAF* mutation frequency.

In conclusion, *MLH1* promoter methylation is a major determining factor for the clinicopathologic and molecular heterogeneity of CIMP-H CRC. In contrast with the typical features of *MLH1*-methylated CIMP-H CRC, which include an older age of onset, a proximal colonic tumor location, mucinous histology, intense lymphoid reactions, frequent *BRAF* mutations, frequent *RUNX3*/*SOCS1* promoter methylation, MSI-H status, and a hypermutated phenotype, *MLH1*-unmethylated CIMP-H CRC is characterized by a middle to old age of onset, a relatively even distribution between the proximal and distal colorectum, less prominent mucinous and lymphoid histologic features, frequent *KRAS* and *APC* mutations, MSS/MSI-L status, and a non-hypermutated phenotype (Figure 4A). Although different serrated pathways to CRC remain to be further understood, our findings suggest that *MLH1*-methylated CIMP-H CRC may be derived typically from the SSA/P pathway but that *MLH1*-unmethylated CIMP-H CRC may develop mainly through the *KRAS*-mutated TSA pathway and also less commonly through the *BRAF*-mutated TSA pathway and/or the SSA/P pathway (Figure 4B).

## MATERIALS AND METHODS

### Case selection

For the present study, 65 CIMP-H CRC samples were selected from our previously established CRC sample cohorts, which consisted of 814 CRC samples in total [28, 29]. All samples from our CRC cohorts were retrospectively collected formalin-fixed paraffin-embedded (FFPE) tissues from 814 patients who underwent surgical resection for CRC at Seoul National University Hospital, Seoul, Korea, between 2004 and 2008. Of the 65 CIMP-H CRCs, 33 were *MLH1*-methylated CIMP-H CRCs, and 32 were *MLH1*-unmethylated CIMP-H CRCs. Based on previous studies [28, 29], by immunohistochemical staining, all 33 of the *MLH1*-methylated tumors were confirmed to have lost MLH1 expression, whereas all 32 of the *MLH1*-unmethylated tumors were confirmed to have retained MLH1 expression. A detailed procedure for CIMP determination, including *MLH1* methylation analysis, is described in the section below. This study was approved by the institutional review board.

### Clinicopathologic data

The clinical and pathologic features of the 65 CIMP-H CRCs were investigated by a review of medical records or by microscopic examination of hematoxylin and eosin-stained FFPE tissue slides. The clinicopathologic parameters included age, sex, tumor location, gross tumor type, American Joint Committee on Cancer (AJCC) tumor-node-metastasis (TNM) stage, disease-free survival, tumor differentiation, mucinous histology, signet ring cell histology, medullary histology, serrated histology, lymphatic invasion, vascular invasion, perineural invasion, tumor budding, tumor-infiltrating lymphocytes (TILs), peritumoral lymphoid reaction, and Crohn-like lymphoid reaction. The mean follow-up time for disease-free survival data of our original cohort (65 patients with CIMP-H CRC) was 36 months (ranging from 1 to 89 months). The detailed assessment criteria for the histopathologic parameters used in this study have been previously described [29, 30]. The number of TILs was counted in one high power field (×400) of the most TIL-intense area. All of the histopathologic parameters were independently evaluated by two pathologists (J.H.K. and J.M.B.). Conflicting assessment results between the pathologists were reviewed and discussed until consensus was reached.

### CIMP analysis

Genomic DNA extraction from 814 CRC FFPE tissues and subsequent bisulfite modification and CIMP analysis of these DNA samples were conducted as previously described [28, 31]. For CIMP determination, methylation-specific quantitative PCR analysis (MethyLight assay) was performed using eight CIMP markers (*MLH1*, *CACNA1G*, *CDKN2A* (*p16*), *CRABP1*, *IGF2*, *NEUROG1*, *RUNX3*, and *SOCS1*). A CpG island locus was defined as hypermethylated when the percentage of the methylated reference value exceeded 4. CIMP-H was defined when a tumor showed promoter CpG island hypermethylation in five or more CIMP markers [32]. CIMP-low (CIMP-L) was defined when a tumor showed promoter CpG island hypermethylation in one to four CIMP markers. CIMP-negative (CIMP-0) was defined when all CIMP markers in a tumor were unmethylated. CIMP-H generally represents the true CIMP-positive status. Of the 814 CRCs subjected to CIMP analysis, 65 were determined to be CIMP-H CRCs and were finally included in this study. Of the remaining 749 cases, 445 were CIMP-L tumors, and 304 were CIMP-0 tumors. Reproducibility of MethyLight assay for quantification of promoter methylation of CIMP markers had been validated by previous data [33]. Performance of the eight-marker panel with a cutoff value of five for determination of CIMP-H in CRC had been validated by our previous study [32].

### MSI analysis

MSI analysis was performed as previously described [28, 31]. In detail, the instability statuses of 65 paired CIMP-H CRC tumor and normal DNA samples were analyzed for the five NCI-recommended microsatellite markers (the Bethesda panel; BAT-25, BAT-26, D5S346, D17S250, and D2S123) using fluorescent multiplex PCR [34]. MSI-H was defined when tumor DNA showed instability (an amplicon length alteration) in two or more microsatellite markers compared with normal DNA. MSI-low (MSI-L) was defined when tumor DNA showed instability in one microsatellite marker. Microsatellite stable (MSS) status was considered when tumor DNA showed no instability in any of the five microsatellite markers. MSI-H generally represents the true MSI-positive status, whereas MSI-L and MSS are considered MSI-negative statuses.

### *KRAS*/*BRAF* mutation analysis

The mutational statuses of *KRAS* codons 12 and 13 and *BRAF* codon 600 were assessed as previously described [28, 31]. Allele-specific PCR and direct sequencing methods were used for these mutation analyses. Of the 65 CIMP-H CRCs, due to the suboptimal quantity or quality of extracted DNA, four samples and one sample were finally excluded from *KRAS* and *BRAF* mutation analyses, respectively.

### TCGA data analysis

Using the publically accessible TCGA data portal (https://tcga-data.nci.nih.gov/tcga/) and TCGA-based publications [35, 36], clinical and molecular data on 36 CIMP-H CRCs were collected and analyzed. The assessed parameters included age, sex, tumor location, TNM stage, histologic subtype (mucinous and non-mucinous adenocarcinomas), lymphatic invasion, vascular invasion, MSI status, mutational phenotype (hypermutated and non-hypermutated; using a cutoff value of 12 mutations per 10^6^ exonic bases) [35, 36], exome-wide mutation rate (including silent and non-silent mutations) [35], and *KRAS*/*BRAF*/*PIK3CA*/*APC*/*CTNNB1*/*TP53* mutations. The differential features according to *MLH1* epigenetic silencing status (silenced *versus* non-silenced) of the CIMP-H CRCs were statistically analyzed.

### Statistical analysis

Statistical analyses in this study were performed using IBM SPSS Statistics version 20 (IBM Co., Armonk, NY, USA) and the R software (http://www.r-project.org/). Comparisons of categorical variables between *MLH1*-methylated (−silenced) and *MLH1*-unmethylated (−non-silenced) tumors were conducted using the chi-square test or Fisher's exact test. Comparisons of continuous variables between *MLH1*-methylated (−silenced) and *MLH1*-unmethylated (−non-silenced) tumors were conducted using the *t*-test. Survival analysis was performed using the Kaplan-Meier method with the log-rank test. All *P*-values were two-sided, and statistical significance was determined at *P* < 0.05.

## SUPPLEMENTARY MATERIAL TABLES AND FIGURES


