# Supplementary material for: Distinct features between MLH1-methylated and unmethylated colorectal carcinomas with the CpG island methylator phenotype: implications in the serrated neoplasia pathway
Source: Oncotarget. 2016 Feb 13;7(12):14095–111. doi: 10.18632/oncotarget.7374 (PMC4924700; doi:10.18632/oncotarget.7374)
Supplement: Supplementary file 1 [file oncotarget-07-14095-s001.pdf]

Distinct features between *MLH1*-methylated and unmethylated colorectal carcinomas with the CpG island methylator phenotype: implications in the serrated neoplasia pathway

Supplementary Material

Supplementary Table S1. Comparison of the frequency of mucinous differentiation between *MLH1*-methylated and unmethylated tumors in proximal-located CIMP-H colon carcinomas (original study cohort; n = 45)

		Case No.	*MLH1*-methylated (n = 27)	*MLH1*-unmethylated (n = 18)	*P*-value
Mucinous histology	Absent	22	8 (30%)	14 (78%)	0.002
	Present	23	19 (70%)	4 (22%)	

Supplementary Table S2. Comparison of the frequency of mucinous differentiation between *MLH1*-methylated and unmethylated tumors in distal-located CIMP-H CRCs (original study cohort; n = 20)

		Case No.	*MLH1*-methylated (n = 6)	*MLH1*-unmethylated (n = 14)	*P*-value
Mucinous histology	Absent	12	1 (17%)	11 (79%)	0.018
	Present	8	5 (83%)	3 (21%)	

Supplementary Table S3. Comparison of the frequency of mucinous differentiation between MSI-H and MSS/MSI-L tumors in CIMP-L/0 CRCs (independent cohort; n = 687)

		Case No.	MSI-H, CIMP-L/0 (n = 47)	MSS/MSI-L, CIMP-L/0 (n = 640)	*P*-value
Mucinous histology	Absent	613	30 (64%)	583 (91%)	< 0.001
	Present	74	17 (36%)	57 (9%)	

Supplementary Table S4. Clinicopathologic features of *BRAF*-mutated, *MLH1*-unmethylated CIMP-H CRCs (original study cohort; n = 4)

Case #	Age	Sex	Tumor location	TNM stage	Tumor differentiation	Lymphatic invasion	Venous invasion	Perineural invasion	Tumor budding	MSI status	Death or tumor recurrence	Disease-free survival
SD-074	52	Female	Ascending colon	Stage IV (pT4N2M1)	Poorly differentiated	Present	Present	Present	Present	MSS	Yes (death)	3.2 months
SD-226	49	Female	Rectum	Stage IV (pT4N1M1)	Poorly differentiated	Present	Absent	Present	Present	MSS	Yes (death)	3.8 months
SD-461	52	Female	Ascending colon	Stage II (pT3N0M0)	Moderately differentiated	Absent	Absent	Present	Present	MSS	No	42.7 months
SD-733	49	Female	Transverse colon	Stage III (pT3N2M0)	Moderately differentiated	Present	Absent	Present	Present	MSS	Yes (recurrence)	19.5 months

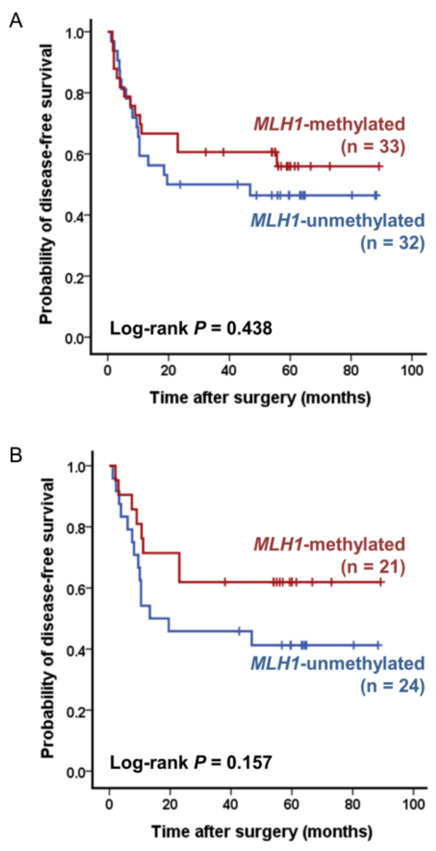

Supplementary Figure S1. Kaplan-Meier survival analysis with log-rank test. **(A)** Survival analysis in 65 CIMP-H CRCs. **(B)** Survival analysis in 45 CIMP-H CRCs treated with 5-FU-based adjuvant chemotherapy.

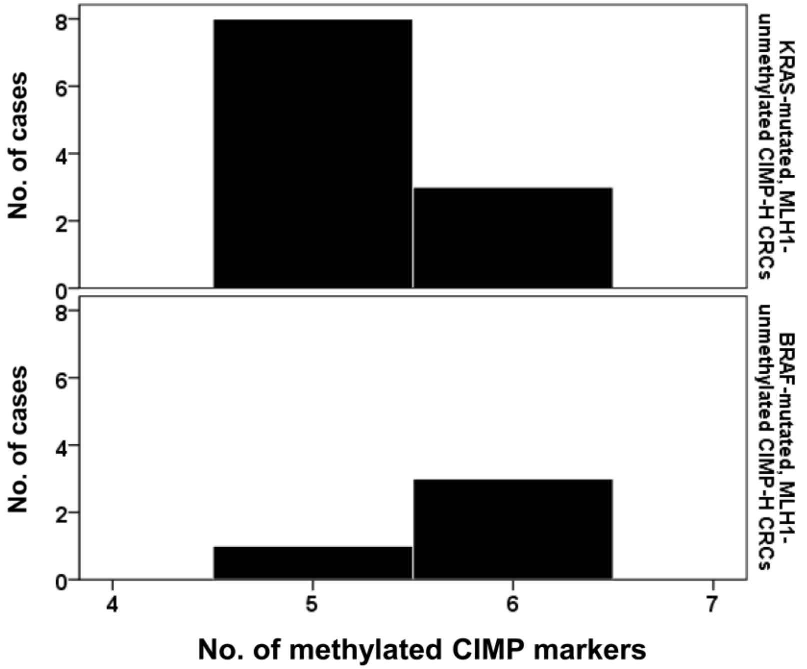

Supplementary Figure S2. Comparison of number of methylated CIMP markers between *KRAS*-mutated and *BRAF*-mutated tumors in *MLH1*-unmethylated CIMP-H CRCs (n = 15).
